# Impact of Chromosomal Architecture on the Function and Evolution of Bacterial Genomes

**DOI:** 10.3389/fmicb.2018.02019

**Published:** 2018-08-27

**Authors:** Thøger J. Krogh, Jakob Møller-Jensen, Christoph Kaleta

**Affiliations:** ^1^Department of Biochemistry and Molecular Biology, University of Southern Denmark, Odense, Denmark; ^2^Institute of Experimental Medicine, Christian-Albrechts-University Kiel, Kiel, Germany

**Keywords:** bacterial nucleoid structure, gene expression, nucleoid associated proteins, genome evolution, chromosomal architecture

## Abstract

The bacterial nucleoid is highly condensed and forms compartment-like structures within the cell. Much attention has been devoted to investigating the dynamic topology and organization of the nucleoid. In contrast, the specific nucleoid organization, and the relationship between nucleoid structure and function is often neglected with regard to importance for adaption to changing environments and horizontal gene acquisition. In this review, we focus on the structure-function relationship in the bacterial nucleoid. We provide an overview of the fundamental properties that shape the chromosome as a structured yet dynamic macromolecule. These fundamental properties are then considered in the context of the living cell, with focus on how the informational flow affects the nucleoid structure, which in turn impacts on the genetic output. Subsequently, the dynamic living nucleoid will be discussed in the context of evolution. We will address how the acquisition of foreign DNA impacts nucleoid structure, and conversely, how nucleoid structure constrains the successful and sustainable chromosomal integration of novel DNA. Finally, we will discuss current challenges and directions of research in understanding the role of chromosomal architecture in bacterial survival and adaptation.

## Introduction

The bacterial chromosome has a free coiling linear length much longer than the average cell and hence requires extensive compaction to fit flexibly inside the cytoplasm (Reyes-Lamothe et al., 2008; De Vries, 2010). For instance, a typical *Escherichia coli* cell 2 μm in length hosts a chromosome composed of 4.6 megabase pairs. Assuming that each base pair occupies 3.4 Å in length, this makes the linear length of the genome 1.7 mm, about 850 times the cell length. It does, however, fit inside the confinements of the cell where it occupies ∼15% of the volume (Reyes-Lamothe et al., 2008). The bacterial chromosome and the structuring proteins are collectively referred to as the nucleoid, which is highly condensed and form compartment-like structures within the cell (Thanbichler et al., 2005a). The specific nucleoid organization is often highly underestimated when considering the fact that bacterial species often excels in quickly adapting to significant changes in the environment (Thanbichler et al., 2005a,b; Thanbichler and Shapiro, 2006; Wiggins et al., 2010).

Excellent reviews have been written on the properties of the bacterial nucleoid, and the forces involved in shaping the nucleoid (Dillon and Dorman, 2010; Dorman, 2013b). In this review, we expand on this topic, with focus on the structure-function relationship of the bacterial nucleoid and how it relates to evolution. A general overview of the fundamental physical and chemical properties that shape the chromosome will be considered in the context of the living cell. Finally, the dynamic living nucleoid will be discussed in the context of evolution. How does acquisition of foreign DNA affect the nucleoid structure, and conversely, how does the nucleoid structure constrain the successful and sustainable chromosomal integration of novel DNA? In the end, we will look at current research gaps and discuss fundamental concepts that emanate from these.

## The Living Nucleoid

The chromosome is composed of DNA and exists *in vivo* in complex with a multitude of proteins that shape the DNA and control its genetic output according to the physiological and environmental conditions of the cell. In this review, we divide the forces that shape the nucleoid into intrinsic and extrinsic forces. Intrinsic forces are physical and chemical properties universal to DNA polymers, whereas extrinsic forces are imposed on the nucleoid by interactions with other macromolecules, such as nucleoid associated proteins (NAPs).

### Intrinsic Forces of DNA

#### Chemical Properties

**FIGURE 1:**
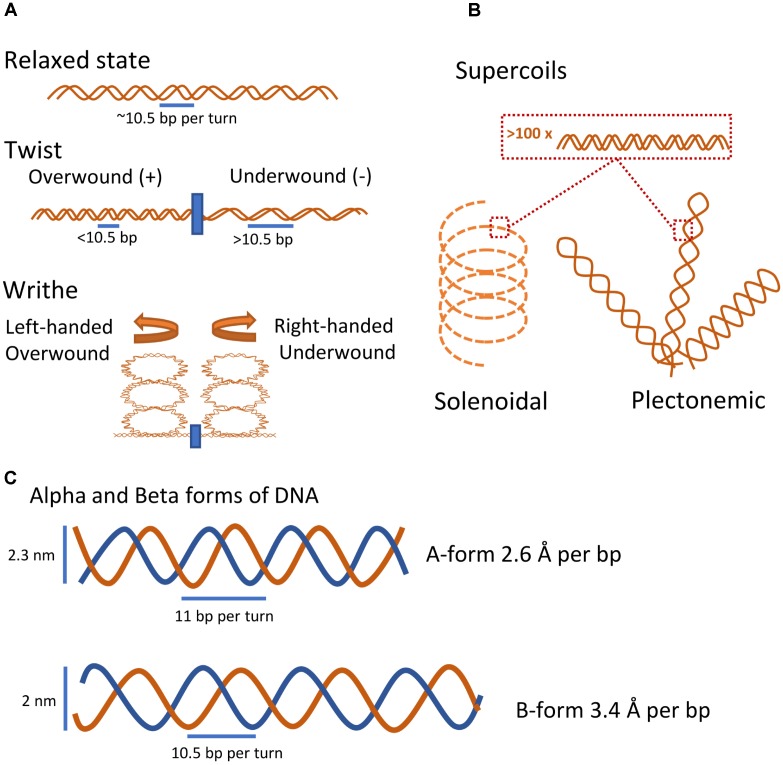
Intrinsic properties of DNA. **(A)** Basic coiled conformational states of DNA. In its relaxed state and under physiological conditions, DNA forms a double helix with 10.5 base pairs per turn. Introducing or removing turns causes the DNA to form local coiled elements either as twists in the linear dimension or by the formation of higher-order writhes (Corless and Gilbert, 2016). **(B)** Coiled conformational states of large DNA fragments. Two major higher-order supercoiled states of DNA, the stable plectonemic state and the less stable but more compact solenoidal state, emerge through extensive over- or under-winding of the DNA helix (Corless and Gilbert, 2016). **(C)** Rough sketch of the two basic molecular forms of DNA. At low hydration levels, DNA will take the A-form, the compact and wider helix form. At physiological conditions, DNA will adopt the more accessible B-form.

Besides being a carrier of genetic information, the DNA sequence ultimately dictates the structural conformation of the nucleoid, directly or indirectly via binding of extrinsic factors (Japaridze et al., 2017). Some of the earliest studies of DNA structure reported that the X-ray diffraction pattern of DNA crystals depended on the hydration level, and the two observed forms were termed Alpha (A-form) and Beta (B-form) (**Figure 1C**; Franklin and Gosling, 1953a,b). The A-form was shown to be 20% shorter than the B-form per base pair, at the expense of a larger diameter (Franklin and Gosling, 1953a,b; Watson and Crick, 1953; Waters et al., 2016). Although the B-form is highly favored *in vivo* due to high water activity, a dynamic equilibrium likely exists between the two DNA structures *in vivo*. GC rich sequences tend to adopt A-form, and the same is true for RNA-DNA and RNA-RNA complexes (Shakked et al., 1989; Waters et al., 2016). Binding of extrinsic factors may also shift the conformation of DNA from B- to A-form due to solvent access inhibition (DiMaio et al., 2015).

Both A and B-form DNA double helices contain minor and major grooves, with the major groove being superior in distinguishing between bases and dominant site for binding of macromolecules that recognize DNA sequences (Franklin and Gosling, 1953a,b; Watson and Crick, 1953; Travers and Muskhelishvili, 2015). However, the grooves of the B-form DNA are more accessible compared to those of the more compact Alpha form (Franklin and Gosling, 1953a,b).

#### Physical Properties

The dynamic helical nature and closed-circular form of prokaryotic chromosomes naturally results in the formation of DNA coiling whereby winding of the polymer around its longitudinal axis and twirling introduces changes in the DNA conformation (**Figure 1A**; Thanbichler et al., 2005a; Gilbert and Allan, 2014). In this review coiling describes local changes to DNA coil (<1.000 bp), whereas supercoiling is used to describe coiling on a major scale (>1.000 bp). Winding in the same direction as the helix will tighten the structure and introduce positive coiling, whereas winding in the opposite direction will loosen the helix and produce negative coiling (Gilbert and Allan, 2014; Corless and Gilbert, 2016). Local introduction of coils in the nucleoid can cause global coils termed supercoils that structure and compartmentalize large parts of the molecule in a sequence-dependent manner (Muskhelishvili and Travers, 2016). Extensive supercoiling will reduce the accessibility of DNA base pairs; however, relatively low levels of negative supercoiling increases the accessibility of the DNA (Gilbert and Allan, 2014). Interestingly, regardless of biological origin, purified closed-circular DNA molecules are almost always negatively supercoiled, and rarely relaxed, indicating that active DNA underwinding occurs *in vivo* (Bauer, 1978).

Two distinct types of DNA supercoiling structures have been described (**Figure 1B**). Plectonemic-type is a broad term applied to any higher-order supercoiling where strands are intertwined in a simple and regular manner. Although plectonemic supercoiling is the most stable and common higher-order DNA structure *in vitro* and *in vivo*, it is not compacting the DNA sufficiently well to solely account for the nucleoid fitting inside the confines of a cell (Thanbichler et al., 2005a,b). Solenoidal type supercoils differ from plectonemic in that they are less stable, but much more compact, due to a tight left-handed turn as opposed to the right-handedness of plectonemic supercoils (Thanbichler et al., 2005a,b). Both types are readily interchangeable and exist in a dynamic equilibrium determined by interaction with DNA binding proteins *in vivo* (Thanbichler et al., 2005b; Corless and Gilbert, 2016). In addition, alternative local DNA structures such as cruciform structures of palindromic sequences, Z-DNA, G-quadruplexes, and opening of the double helix into single-stranded DNA have been reported. These specialized structures are induced by negative supercoiling, thus underlining the ability of DNA supercoiling to regulate the nucleoid structure on a local and global scale (Mizuuchi et al., 1982; Gilbert and Allan, 2014; Corless and Gilbert, 2016).

DNA is not an inert macromolecule, but rather a highly flexible macromolecule that changes shape and conformation depending on physiological conditions in a somewhat sequence dependent manner. The shape is however just the first tier of complexity; both the specific sequence, structure, and strandedness of the DNA can facilitate interactions with extrinsic factors that in turn may induce conformational changes, which may further regulate the binding of other extrinsic factors (Shakked et al., 1989; Travers and Muskhelishvili, 2015). Theoretical and experimental research suggests that DNA *in vivo* behaves as cross-linked DNA polymer gel rather than free coiling DNA, indicating that the nucleoid interacts extensively with extrinsic factors (De Vries, 2010; Cagliero and Jin, 2013). *In vivo* supercoiling is further highly influenced by binding of extrinsic protein factors to the DNA (Gilbert and Allan, 2014).

### Extrinsic Forces

Having no specific compartment within the highly crowded cytoplasm, the nucleoid is free to interact with a plethora of enzymes, proteins, metabolites, and other molecules throughout the cell cycle. All these extrinsic factors influence the nucleoid structure and are influenced by the interaction themselves. Of these factors topoisomerases that regulate DNA supercoiling levels are of particular interest.

#### Topoisomerases and Supercoiling

Topoisomerases work by either nicking one strand in the DNA and let it rotate around the non-cut DNA strand (Type 1), or by cutting both strands of the DNA and passing another part of the DNA through this gap (Type 2) (Bush et al., 2015). Two topoisomerase enzymes are essential for DNA replication progression and/or DNA decatenation: DNA gyrase (Topoisomerase II) and Topoisomerase IV (Bush et al., 2015). The DNA gyrase, or topoisomerase II, is an ATP-dependent Type 2 topoisomerase that generates negative supercoils in the nucleoid and is important for the removal of positive supercoils in front of the replication forks. Thus, DNA gyrase is heavily involved in maintaining chromosomal supercoiling levels (Schvartzman et al., 2013). Topoisomerase IV is another ATP-dependent Type 2 topoisomerase essential for decatenation of newly replicated DNA before chromosomal segregation (Schvartzman et al., 2013). It relaxes both negative and positive supercoiling, the latter at a 20-fold faster rate, but is unable to introduce supercoiling as DNA gyrase (Schvartzman et al., 2013; Bush et al., 2015). Besides these enzymatic interactions, which actively manipulate DNA supercoiling levels, a plethora of nucleoid-structuring proteins bind to the nucleoid and modulate its structure through DNA bending rather than active opening and twisting of the DNA. These nucleoid-structuring proteins can generate- and to some extent restrict supercoiling through simple DNA binding events.

#### Nucleoid Associated Proteins

Proteins that modulate the nucleoid structure are collectively referred to as NAPs. They have different recognition capabilities that identify binding sites by sequence specificity, through recognition and interaction with specific bases, and/or structural specificity, through interaction with the phosphate-backbone structure (Duzdevich et al., 2014). In general, the NAPs are highly conserved within specific bacterial families, but a few are highly conserved among all prokaryotic species, suggesting a fundamental advantage of nucleoid structuring that has been further improved in the course of evolution. All bacterial species encode at least one NAP (Dorman, 2014).

The binding properties of NAPs are fundamental to their function. By binding to the nucleoid, the NAPs can bend the DNA and thereby bring distant domains into proximity, or disrupt existing structures (Macvanin and Adhya, 2012; Lioy et al., 2018). NAPs can further connect two distinct parts of the nucleoid through binding at two or more positions followed by bridge formation (Song and Loparo, 2015). In general NAPs have little to no sequence-specific DNA recognition, structural recognition may, however, possibly result in sequence composition specific recognition. Many of them do have a preference for AT-rich regions (Lang et al., 2007; Gordon et al., 2011), possibly due to a higher requirement for regulation and structuring of these regions due to the intrinsic instability of AT-rich DNA. NAP binding to AT-rich promoter regions also facilitates transcriptional regulation of horizontally acquired genes that are often AT-rich (Lucchini et al., 2006; Gordon et al., 2011). One intriguing example of transcriptional regulation is performed by the histone-like nucleoid structuring protein H-NS. Polymers of H-NS often bind large AT-rich regions of DNA, such as horizontally acquired DNA, and thus inhibit transcription of these regions (Lucchini et al., 2006). Transcription from neighboring DNA regions can, however, disrupt local H-NS repression (Wade and Grainger, 2018). Interestingly, *Salmonella typhimurium* H-NS knockouts exhibit severely reduced growth rates, rescuable in combination with mutations to or inactivation of the stress response sigma factor RpoS, suggesting a coordinated response of H-NS and RpoS under stress (Lucchini et al., 2006, 2009). Another property of NAP binding is the containment of supercoiling it may impose (Higgins, 2016). Intriguingly, in *E. coli*, the specific NAP encoding genes are positioned on the chromosome according to the abundance of the NAPs relative to growth phase, with genes for NAPs highly expressed during optimal growth, positioned closer to the origin, and genes for NAPs highly expressed during late stage growth, positioned closer to the terminus, suggesting a spatiotemporal gene expression pattern for NAPs during the bacterial growth cycle (Sobetzko et al., 2012; Japaridze et al., 2017).

**FIGURE 2:**
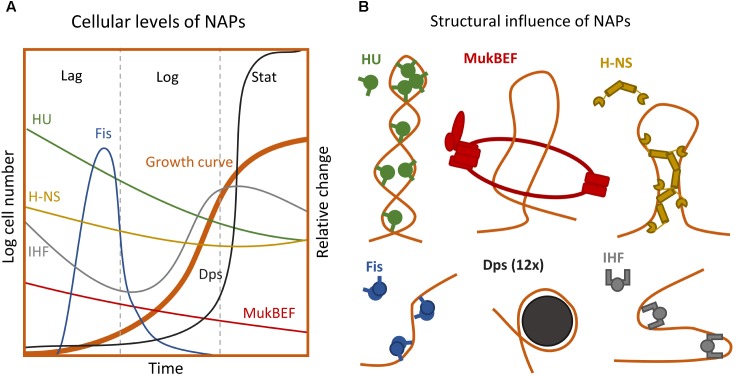
Extrinsic forces influencing the chromosome structure. **(A)** Intracellular levels of the major NAPs during growth phases (Not to scale) (Talukder and Ishihama, 1999, 2015). **(B)** Impact of different NAPs on the nucleoid superstructure (Badrinarayanan et al., 2015; Winardhi et al., 2015; Yamanaka et al., 2018). For detailed description of structural impact see **Table 1**.

**Table 1** and **Figure 2** contain general information about some of the most abundant and well-known NAPs from *E. coli*. Certain NAPs, such as HU, are conserved among almost all prokaryotes; others, such as H-NS, Fis, and Dps, are found only in *E. coli* and related enterobacteria (Dillon and Dorman, 2010).

**Table 1:** General information of the most abundant and well-known NAPs in *Escherichia coli* (Talukder and Ishihama, 1999; Dillon and Dorman, 2010; Dorman, 2014; Badrinarayanan et al., 2015; Lioy et al., 2018; Yamanaka et al., 2018).

Name	Size (Mono)	Stoichiometry	Gene(s)	DNA binding preference	Impact on DNA structure	Conservation
HU – ‘Histone-like protein from *E. coli* strain U93’ or ‘Heat-unstable nucleoid protein’	18 kDa	Homo/heterodimer	*hupA* (α) and *hupB* (β)	No sequence specificity, but preferably AT-rich DNA	Sharp bends/loops Higher-order complexes that structures and stabilize the DNA helix of the nucleoid. Long-range interactions	At least one subunit present in most bacterial genomes
H-NS – ‘Histone-like Nucleoid Structuring protein’	15.5 kDa	Homodimer/polymer	*H-NS*	No sequence specificity, but preferably AT-rich DNA	Bridging between relative distant DNA molecules. Restriction of short-range interactions	Highly conserved within *E. coli* and related strains
Fis – ‘factor for inversion stimulation’	11.2 kDa	Homodimer	*fis*	No sequence specificity, but preferably AT-rich DNA	50–90° bends	Conserved among Enterobacteriaceae
IHF – ‘integration host factor’	11.4 kDa	Heterodimer	*ihfA* and *ihfB*		160–180° bends	Related to HU, but not as conserved
Dps – ‘DNA binding protein form starved cells’	18.7 kDa	Ferritin-like mono/dodecamer	*dps*		Induces crystalline like stable DNA state, protecting the DNA	
MukB (SMC homolog) – ‘structural maintenance of chromosomes’	170 kDa	Homodimer; acts in complex with MukE and MukF	*mukBEF*		Condensin-like. Ring-like structure encircling DNA and forming topologically isolated DNA loops. Long-range interactions	Conserved in gamma-proteobacteria

#### Elucidation of Global Nucleoid Structure

The most frequently used method for determining chromosomal structure is variants of Chromosomal Conformation Capture (3C). Using crosslinking coupled with ligation and subsequent site detection, this method makes it possible to determine which chromosomal positions that are bridged into proximity. This way it is possible to determine a rough map of relative spatial distance between specific nucleoid positions. For a review on methods investigating chromosomal interactions, see Davies et al. (2017). In addition, many studies have used fluorescently tagged NAPs or DNA-binding fluorophores to determine the physical structure of the nucleoid as well as its subcellular localization and condensation level (Woldringh et al., 1994; Talukder and Ishihama, 1999; Cabrera and Jin, 2003).

**FIGURE 3:**
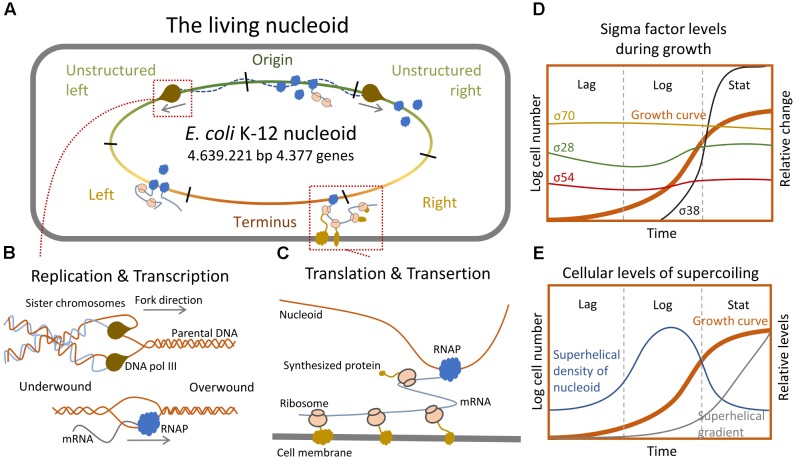
Informational relay and the nucleoid structure. **(A)** Simplified representation of the *Escherichia coli* chromosome and its interactions with the DNA replication- and transcription machineries. **(B)** Sketch of how replication and transcription impact nucleoid structure through changes in supercoiling levels (Corless and Gilbert, 2016). **(C)** Depiction of how translation and transertion affects nucleoid structure by pulling the nucleoid toward the membrane (Bakshi et al., 2015). **(D)** Relative changes in the cellular level of individual sigma factors during distinct growth phases (Gruber and Gross, 2003). **(E)** Cellular levels of supercoiling and emergence of superhelical gradient across the nucleoid during distinct growth phases (Lal et al., 2016).

The nucleoid of *E. coli* has been investigated extensively and has been shown to assume a defined structure and relative position inside the cell (Valens et al., 2004; Espeli et al., 2008; Dupaigne et al., 2012; Cagliero et al., 2013). Based on 3C and DNA-DNA interaction studies, four structured macrodomains of the nucleoid have been identified (Valens et al., 2004; Lioy et al., 2018). These domains are the Origin, Right, Terminus, and Left domains (**Figure 3A**). Two additional non-structured domains, called the non-structured (or mixed) right and left domains, exist between the origin and flanking regions on both sides, respectively. In *E. coli*, an investigation into the global supercoiling pattern revealed that a gradient of negative supercoiling exists in stationary phase cells with the terminus being the most negatively supercoiled. This gradient is absent in exponential phase cells (**Figure 3E**; Lal et al., 2016). Furthermore, the super-helical density of the nucleoid, its compaction level, and the relative positioning of specific domains is highly dynamic and highly dependent on the growth phase (**Figure 3E**). These observations indicate that the state of supercoiling could be affected by the activity of informational flow, i.e., by DNA replication and transcription processes (Hadizadeh Yazdi et al., 2012; Dorman, 2013a,b; Kleckner et al., 2014; Talukder and Ishihama, 2015).

### Informational Relay and Its Relation to the Nucleoid Structure

#### Replication and Supercoiling

Common for all processes related to the flow of genetic information – whether it is the replication or gene expression – is that they in some way affect nucleoid conformation (**Figures 3A,B**; Crick, 1970). Genome replication is carried out by the replisome consisting of several different enzymes including DNA polymerase III (Kelman, 1995). During this process, the DNA must be opened to allow Watson-Crick base pairing between newly incorporated nucleotides and their parental strand templates (Corless and Gilbert, 2016). Since the replication machinery is too big to rotate along the DNA helix quickly enough to account for the fast progression of DNA synthesis, it forces the upcoming un-replicated DNA to be tightened, thus creating positive supercoiling ahead of the replication fork. Conversely, negative supercoiling builds up in the newly synthesized semiconserved DNA tailing the replication fork (Keszthelyi et al., 2016). This introduces two problems: on one hand accumulation of positive supercoiling in front of the replication fork eventually blocks the replisome progression and, on the other hand, excess negative supercoiling trailing the fork causes the newly synthesized dsDNA strands to catenate and thereby hinders chromosome segregation (Joshi et al., 2013). To prevent these lethal scenarios the bacterium utilizes topoisomerases to control supercoiling levels throughout the cell cycle.

**FIGURE 4:**
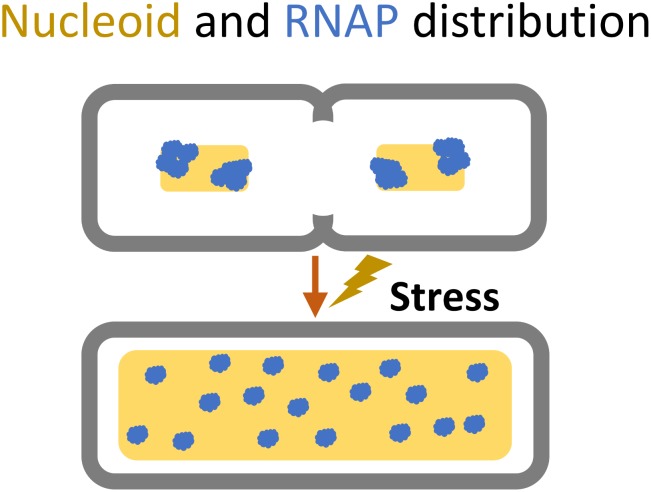
Cellular space occupied by the nucleoid (yellow) and the dispersion of RNA polymerase (blue) during shift from rapid growth to slow or stressed growth.

Chromosome replication initiates at one spot, the origin of replication (*oriC*) (Wolanski et al., 2015). Initiation of replication relies on the interaction of several NAPs at the *oriC* sequence; HU, FIS, and IHF are known to stimulate initiation, and DnaA is required to open the DNA duplex (Wolanski et al., 2015). The replication machinery progresses along the length of the nucleoid on both sides in a bidirectional manner and terminates replication at the terminus macrodomain, followed by segregation of the sister nucleoids (Wang et al., 2013b). During rapid growth, the nucleoid is highly condensed, and each cell can have several overlapping replications cycles taking place simultaneously, thus creating differences in gene copy number depending on their genomic proximity to the origin of replications (Wang and Rudner, 2014). Upon transition to stress or slower growth the nucleoid relaxes and expands into the entire cytoplasm, and the gene copy ratio between origin proximal- and terminus proximal genes approaches one (**Figure 4**; Wang and Rudner, 2014).

How nucleoid domains are positioned in the cell during replication has been determined in multiple bacterial species including *E. coli* (Wang and Rudner, 2014). During slow-growth, the nucleoid adopts a transverse organization, where the origin is positioned at mid-cell, with the structured left and right regions flanking either side, respectively, and the terminus connecting these flanks. Before cell division the two sister nucleoids retain this organization but are segregated to either side of the septal plane at mid-cell (Wang and Rudner, 2014). At fast growth, the nucleoid adapts another organization where the newly replicated origins relocate to the cell poles, leaving the replication machinery and non-replicated nucleoid positioned between them. This creates a pattern where just before cell division either cell half will contain a nucleoid copy arranged with the origin of replication near or at the pole, and with the right and left regions spanning next to each other to the terminus region located at mid-cell (Wang and Rudner, 2014).

Intriguingly, the structure of origin-proximal nucleoid domains has been shown to depend on the position of replication origin rather than the DNA sequence of the domains, suggesting that replication impacts structured and non-structured regions near the origin- and terminus irrespective of their nucleotide sequence (Duigou and Boccard, 2017).

#### Transcription, Supercoiling and Stress

Transcription affects DNA topology in much the same way as DNA replication, as the transcribing RNA polymerase (RNAP) introduces positive supercoiling immediately downstream of the transcription complex and negative supercoiling upstream of it (**Figures 3A,C**; Ma et al., 2013). The link between transcription and nucleoid compaction is further supported by observations that negatively supercoiled genes are generally more efficiently transcribed than compact positively supercoiled genes, due to increased exposure of a looser promoter region more prone to the opening (Sobetzko et al., 2013; Gilbert and Allan, 2014). This two-way transmission might act as a feedback loop, in which the transcribing RNAP induces further transcription via generation of negative supercoiling in its wake. Indeed supercoiling is generally considered a transcriptional regulator (Dorman, 2013b). Genes that are highly affected by supercoiling represent ∼7% of the gene pool. They are generally AT rich and dispersed throughout the entire genome with no apparent pattern (Peter et al., 2004).

The transcription machinery is composed of the core RNA polymerase and a variable Sigma Factor. The core RNAP enzyme is composed of 5 subunits (α_2_ββ’ω) and is capable of non-specific DNA binding (Saecker et al., 2011). To initiate RNA synthesis, RNAP requires either DNA ends/nicks or assembly of the RNAP core enzyme and a sigma factor into a Holoenzyme (Saecker et al., 2011). However, before the holoenzyme can initiate RNA synthesis, it searches the nucleoid for promoters in a three dimensional and non-sequence specific manner until it recognizes a promoter region, specified by the sigma factor associated with the holoenzyme (Svetlov and Nudler, 2013; Wang et al., 2013a; Duzdevich et al., 2014). All bacteria have at least one housekeeping sigma factor for recognition of promoters associated with normal growth in a non-changing and optimal environment, and in *E. coli* this sigma factor is called Sigma70 (RpoD or SigmaD). Six other Sigma factors have been identified in *E. coli*, each recognizing promoters of genes related to some specific environmental condition, e.g., lack of iron (Sigma19/FecI), heat stress (Sigma 32/RpoH), extracytoplasmic stress (Sigma 24/RpoE), motility (Sigma 28/RpoF), nitrogen metabolism (Sigma 54/RpoN), and the sigma factor related to starvation/stationary phase or general stress (Sigma38/RpoS) (Maeda et al., 2000).

Neither RNAP nor sigma factors can bind promoter DNA selectively. Thus, the RNAP requires the sigma factor to bind to a specific promoter, and sigma factors do not occupy promoters without RNAP (Gruber and Gross, 2003). Sigma factors compete for RNAP binding, and the binding affinity and number of molecules present at a given time depends on the environment and status of cell growth (**Figure 3D**; Sharma and Chatterji, 2010; Peano et al., 2015). *E. coli* cells contain approximately 2.000 RNAP core enzymes during normal growth (Maeda et al., 2000), and the amount of RNAP decreases to approximately 65% in stationary phase thereby increasing the competition among the sigma factors for RNAP (Nyström, 2004). The transcriptional speed of a gene correlates with its functional importance in response to changing environments. Genes encoding central regulators have high transcriptional speeds, and are highly influenced by DNA topology and codon composition (Großmann et al., 2017). The nucleoid distribution of RNAP is highly dependent on the growth condition. In rapidly growing cells, the polymerase is localized to distinct transcriptional foci, whereas the distribution is equalized over the entire nucleoid at slow growth (Cabrera and Jin, 2003; Cagliero and Jin, 2013; Gaal et al., 2016). This suggests active clustering of highly expressed genes. Consistent herewith, recent 3C studies showed a proportional relation between transcriptional level of genes and their contact frequencies (Lioy et al., 2018). The ability of RNAP to diffuse through- and attach to the nucleoid is highly affected by its condensation level, and active transcription in turn condenses the nucleoid through supercoiling, thus creating a negative feed-back loop which can be alleviated by the topoisomerases (Cabrera et al., 2009; Corless and Gilbert, 2016). Intriguingly, in a ChIP-seq experiment it was observed that 23% of all promoter-bound RNAP-Sigma 70 holoenzymes during exponential growth at optimal growth conditions were transcriptionally inactive (Reppas et al., 2006). These non-active RNAPs were positioned at promoters with large differences in DNA melting temperature (Tm) between the promoter and the corresponding coding sequences, suggesting that an energetic barrier blocks promoter escape. However, the data suggest that other factors are needed to account for the poising of RNA polymerase at these particular sites (Reppas et al., 2006). Similar observations have been made regarding Sigma 38-associated holoenzymes (Peano et al., 2015). Perhaps these poised RNAP function as quick response to environmental changes, both for transcription of important genes and for inducing changes to the nucleoid structure at strategic positions.

#### Translation/Transertion and Nucleoid Structure

The last step in the informational flow involves the translation of the RNA blueprints into amino acid polymers, proteins, by the ribosome. Translation is not physically related to the nucleoid, and thus has a relatively little direct impact on the nucleoid structure. However, in prokaryotes, initiation of translation is possible as soon as the ribosomal binding site has been transcribed, with multiple ribosomes attaching and initiating translation whenever the ribosome binding site is free (Cabrera et al., 2009). Furthermore, membrane protein transertion creates a pull on the nucleoid forcing any actively transcribed region with membrane-associated proteins to be located near the inner membrane, thereby de-condensing the nucleoid (**Figures 3A,C**; Cabrera et al., 2009). This force is considered the major expansion force of the nucleoid, and accordingly, the addition of chloramphenicol to rapidly growing cells will cause the nucleoid to quickly contract due to the halting of translation (van Helvoort et al., 1996; Zimmerman, 2002; Bakshi et al., 2015). Translation also works as a compaction force during normal growth. The ribosomal subunits 30S and 50S mix freely with the nucleoid whereas assembled 70S ribosomes tend to segregate away from the nucleoid (Bakshi et al., 2015). Newly transcribed mRNA inside the dense nucleoid will attract ribosomal subunits, which diffuse into the nucleoid where they assemble into 70S-polysomes that slowly diffuse out of the condensed nucleoid due the osmotic forces (De Vries, 2010; Bakshi et al., 2015). Outside the nucleoid, the existence of ribosome-rich regions suggests that assembled ribosomes compacts the nucleoid by macromolecular crowding (Marenduzzo et al., 2006; Bratton et al., 2011; Bakshi et al., 2015; Pereira et al., 2017).

It appears that there is interdependency between processes mediating the flow of genetic information and the nucleoid topology-status mediated via supercoiling on a local and global scale. NAPs influence this dynamic relation by retaining or obstructing the introduction of supercoils and thus indirectly influence the nucleoid accessibility to DNA- and RNA polymerases. Indeed, NAPs confer structure to the nucleoid and can induce informational flow by bending and opening the nucleoid, or hinder informational flow by simply engulfing the nucleoid (Dillon and Dorman, 2010; Badrinarayanan et al., 2015).

### Bacterial Stress Response – At a Glance

A key mediator of cellular stress is (p)ppGpp, a signal molecule that binds to RNAP and inhibits transcription of promoters with intrinsically unstable open complexes, induces production of the stress sigma factor Sigma 38 by association to the *RpoS* mRNA, and induces RNAP association with the alternative sigma factors. (p)ppGpp further modulates Sigma 70-associated holoenzymes to target maintenance and stress defense related genes and neglect household genes (Nyström, 2004; Wade and Struhl, 2008; Sharma and Chatterji, 2010). During exponential growth, most of the RNA polymerases are distributed at distinct loci where the genes relevant to fast growth, such as rRNA genes, meet in the 3rd-dimensional space (Cagliero and Jin, 2013). But when cells are exposed to stress the RNAP distribution shifts to cover the entire nucleoid (**Figure 4**; Cabrera and Jin, 2003). Upon entry into nutrient stress the cellular levels of the different NAPs also change (Talukder and Ishihama, 1999). HU and H-NS decrease two to three-fold (compared to rapid growth), IHF increases three-fold in transition but decreases two-fold in late stationary, Fis levels drop drastically, whereas Dps is vastly induced (110×) (**Figure 2A**; Talukder and Ishihama, 1999). The changes in NAP levels are consistent with the observed nucleoid decondensation during the transition into stress and enable the RNAP to redistribute to other available DNA regions (**Figure 4**; Talukder and Ishihama, 1999; Hadizadeh Yazdi et al., 2012). At late stationary phase the nucleoid condenses into a crystal-like complex induced by Dps (Badrinarayanan et al., 2015).

**FIGURE 5:**
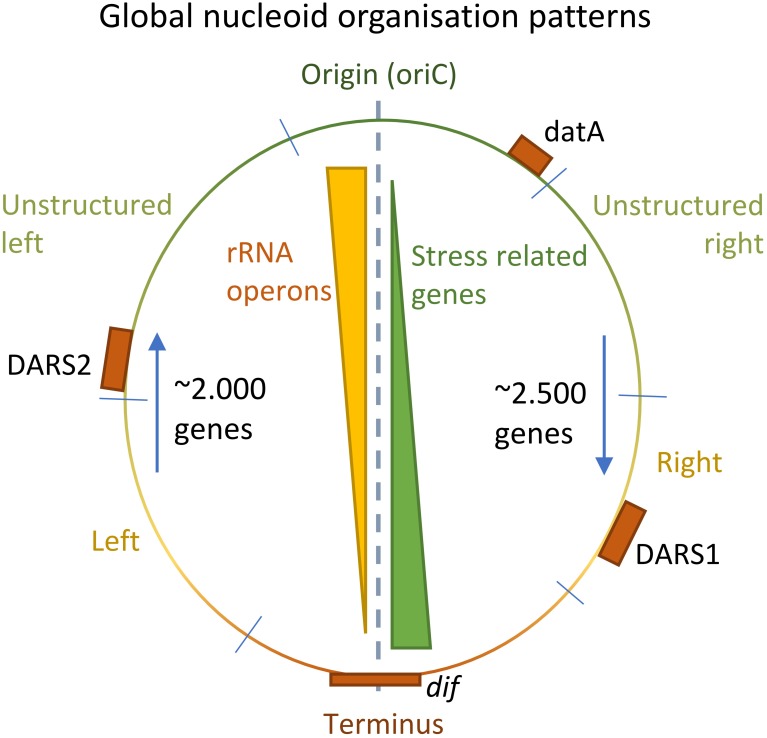
Global patterns of nucleoid organization. Positional gradients of rRNA operons and stress related genes on the genome of *Escherichia coli* (Yellow/Green triangles). Each half of the nucleoid is approximately of equal lengths, and the relative position of the DARS1/2 and datA elements to oriC is conserved among 59 highly different *E. coli* strains (orange boxes) (Frimodt-Møller et al., 2015, 2016; Riber et al., 2016). Furthermore, the number of genes transcribed against the replication fork is lower than the number of genes along the direction of the replication fork (blue arrows).

An intriguing correlation appears to exits between the position of genes on the chromosome relative to the origin, and their involvement in stress responses. Whereas major house-keeping genes such as rRNA operons are located in the origin proximal part of the chromosome, genes involved in stress are predominantly located near the terminus (**Figure 5**; Sobetzko et al., 2012). This organization might be due to the high ori/ter ratio that exists between gene-number in exponential growth but not under stressed non-growth conditions. The organization may further be related to the gradient of negative supercoiling that exists in stationary growth but not in exponential growth (Lal et al., 2016). These examples show the tight connection between the dynamics of the nucleoid structure adaptation to stress.

## The Evolving Nucleoid

### Chromosomal Sequence Variation

Due to the natural force of evolution, life is in a never-ending arms race where organisms that are fitter in an environment will prevail in comparison to those less fit. To survive new environments and types of stress, a prokaryote must evolve, and this generally involves changes to the existing genomic sequence or the acquisition and integration of new DNA. Given the relationship between structure and sequence described above, changes to the chromosome sequence may potentially alter the nucleoid structure, and hence the genetic output, which ultimately determines the fitness-cost associated with the DNA sequence perturbation.

The most basic form of genome alteration is the random introduction of errors during replication, which often has no effect on the genetic output and little or no impact on nucleoid structure. During DNA repair, large changes to the genome may occur through recombination and potentially impose major changes on the nucleoid structure. Double-stranded breaks in the DNA often occurs during a bacterial lifecycle, and errors during the natural recombination may enable chromosomal regions rearrange, i.e., to switch position or become inverted, deleted, or duplicated (Periwal and Scaria, 2015). These changes may severely alter the genomic structure, which in most cases will be detrimental to the cell as only specific regions tolerate inversions and/or change of location (Periwal and Scaria, 2015).

#### Acquisition of Foreign DNA

The ability of bacteria to acquire DNA from the environment through horizontal (or lateral) gene transfer (HGT), is an important means of generating natural variation in prokaryotes (Ochman et al., 2000; Popa and Dagan, 2011). Genes that are not part of the house-keeping or essential gene pool are often referred to as the mobilome, which is accessory genes, since they often originate from a horizontal host rather than a vertical ancestor (Siguier et al., 2014). The impact of HGT on the genomic composition is high. A study estimated 18% of genes in *E. coli* MG1655 have been horizontally acquired since the divergence from *Salmonella* even though *E. coli* is not naturally competent (Ochman et al., 2000; Popa and Dagan, 2011).

Acquisition of novel DNA is however not sufficient by itself to produce a sustainable or competitive new genotype. The foreign DNA needs to be integrated as well, since microbes tend to remove non-functional or unnecessary DNA from the genome (Chih-Horng and Howard, 2008; Popa and Dagan, 2011). Intriguingly, in *E. coli* K-12 two gene classes exist, which together comprise more than 225 genes (∼5% of the total gene pool), with no apparent function at all (Keseler et al., 2013). These are Phantom-genes, which have been identified as genes through computational searches, but without any observed expression, and Pseudo-genes, which are genes with homology to functionally expressed genes, but with changes in their sequences that prevent expression (Keseler et al., 2013; Goodhead and Darby, 2015). The widespread appearance of such genes might indicate that they are not subject to deselection even though they are redundant. Instead their presence at specific positions might be important for a genomic structure that is favorable for the bacterium.

### Integration of Foreign DNA

Optimal integration of horizontally acquired DNA requires positioning of the acquired DNA at the most ideal genomic location for highest fitness in the given environment. Many variables have been shown to influence the successful integration of DNA, such as nucleotide sequence, NAP binding, gene function and expression level, and several positional biases have been observed as well. Uptake of plasmids tends to be a metabolic burden for a cell (Baltrus, 2013), whereas incorporation of whole bacterial genomes into other organisms possibly have minimal large-scale consequences to fitness (Itaya et al., 2005).

#### Impact of Sequence Composition and Function on Integration

The sequence of the acquired DNA is not only important for acquisition but also for sustainable integration into the chromosome. When considering the promoter and regulatory region of foreign DNA, genes that fit into existing regulatory networks are usually retained with higher efficiency. The same is true for genes with similar GC content as the recipient chromosome (Popa and Dagan, 2011). The coding sequence of the newly acquired DNA might have a different codon usage as well, further imposing a strain on the host. Studies, however, suggest that the strain imposed by suboptimal codon usage is highly overrated (Popa and Dagan, 2011; Großmann et al., 2017; Porse et al., 2018).

Horizontally acquired DNA often has a higher AT-content compared to the remaining host genome, and in *E. coli* and related species this is exploited by silencing horizontally acquired DNA through H-NS binding (Navarre et al., 2006; Stoebel et al., 2008; Gordon et al., 2011). H-NS binding has been proposed as a mechanism for avoiding the unwanted expression of newly acquired genes that have yet to be integrated into existing cellular processes (Doyle et al., 2007; Paytubi et al., 2013; Dorman, 2014). This makes it possible for bacteria to obtain e.g., large pathogenicity islands that would otherwise represent a massive burden. The foreign DNA might then first be expressed under a particular stress condition where the new gene products are needed. This idea is further underlined by the observations that high expression levels impact the transferability of a gene negatively (Park and Zhang, 2012). Furthermore, large AT-rich plasmids/xenogeneic DNA elements may disturb the intracellular equilibrium of H-NS regulation by introducing new binding sites. Consistently, many plasmids carry their own H-NS homolog to keep cellular status quo and not decrease the fitness of the host cell severely (Doyle et al., 2007; Takeda et al., 2011). Observations in *Salmonella*, however, indicate that the presence of whole or truncated H-NS homologs in horizontally acquired genetic islands may antagonize the silencing effects of the native H-NS (Walthers et al., 2007, 2011; Cameron and Dorman, 2012). Genes encoding other NAPs, such as Fis and HU, have also been observed on plasmids, further suggesting the importance of NAP equilibrium for minimal fitness cost during successful DNA acquisition (Takeda et al., 2011). H-NS may further facilitate sequence diversification as H-NS-associated genomic regions tend to differ more between closely related species compared to regions without H-NS, but only when these H-NS-associated regions are located upstream of genes (Higashi et al., 2016). Acquisition of foreign DNA may further influence the nucleoid structure through the regulation of H-NS. Compact spatial clusters form through oligomerization of DNA bound H-NS, sequestering the associated operons, and juxtaposing various DNA segments, and deletion of H-NS causes major chromosomal reorganization (Wang et al., 2011 Science; Winardhi et al., 2015; Helgesen et al., 2016). Thus, by association to H-NS, newly inserted AT-rich DNA may juxtapose entire segments of DNA, making even small insertion capable of causing major structural changes.

The function of the foreign DNA sequence also impacts its ability to integrate. If a transferred gene originates from a very distantly related bacterial species, the change in cellular context and possible lack of chaperones can result in a higher protein misfolding rate, which can be lethal for the host (Baltrus, 2013). Horizontally transferred genes are mostly related to the peripheral metabolism (uptake) rather than central metabolism, and rarely to the flow of genetic information. Genes that specify subcomponents of larger cellular complexes are less well integrated too, since they require transfer of all complex-related genes *en bloc* to function properly (Cohen et al., 2011; Popa and Dagan, 2011). Optimal integration does not depend exclusively on the sequence of the integrated DNA, but also on the genomic location of integration. The gene products specified by the foreign DNA need to be functionally integrated into the cellular processes of the recipient host, and in this respect the site of integration on the nucleoid play an important role since both the gene expression level and distribution of gene products may depend on genomic position (Kuhlman and Cox, 2012; Pulkkinen and Metzler, 2013; Bryant et al., 2014).

#### Impact of Genomic Position on Integration

On a global scale, horizontally acquired DNA is evenly distributed among the two halves of the nucleoid, relative to *oriC* and terminus, but also between different regions of the two arms, suggesting a need for conservation of the overall nucleoid proportions (**Figure 5**; Frimodt-Møller et al., 2015). This imposes global constraints to an optimal positional integration of foreign DNA in the genome. However, more local constraints seem to exist at the macrodomain level as well. Moreover, rRNA operons appear to cluster closer to the origin in *E. coli* (**Figure 5**). The directionality of genes is also biased. In *E. coli* K-12 MG1655, approximately 2.000 genes are expressed against the direction of the oncoming replication fork, whereas approximately 2.500 genes are expressed in the direction of a working replication fork (**Figure 5**). This bias might be due to replication fork stalling induced by collisions with the transcription machinery (Sankar et al., 2016).

On a local scale, genes whose products function together in cellular pathways tend to cluster (Ma and Xu, 2013). Transcription of genes will affect neighboring genes through the supercoiling gradient unless supercoiling is released or physical barriers that restrict the transmission of supercoiling are formed. Evidence of supercoiling release has been observed at specific REP elements located at the end of certain open reading frames. REP elements are recognized by the DNA gyrase to enable quick relief of the positive supercoil introduced by RNAP (Dorman, 2014). Barriers restricting the transmission of DNA supercoiling can be formed by binding of NAPs (Rogozin et al., 2002; Meyer and Beslon, 2014). For instance, the nucleoid binding protein HU has been shown to mediate transcriptional insulation (Berger et al., 2016).

Regulation of gene expression by H-NS depends on the chromosomal position of the target promoter, suggesting that spatial distance between regulator and target might have an impact on the level of interaction (Brambilla and Sclavi, 2015). The small amount of non-coding DNA in prokaryotic genomes, and the three-dimensional diffusion of RNAP further advocates for a spatial impact on gene integration.

## Outlook

The nucleoid is central to the life of prokaryotes. As such, much attention has been devoted to the study of the functional properties of the nucleoid as well as its interactions with associated proteins (Dillon and Dorman, 2010; Dorman, 2014). Less is known about the global and domain-level structure dynamics of the nucleoid, and it is often stated in general literature that there is no major organization of the hereditary material in prokaryotes (Reece and Urry, 2011). There is paucity in our understanding of how local (10–100 kbp) nucleoid structure dynamics influences coordination of gene expression and how this impacts the sustainable integration of foreign DNA and hence how bacterial genomes evolve in nature. During growth, many processes take place simultaneously, including replication, segregation, transertion, and transcription, which places a high demand on cellular logistics. Modulation of the nucleoid structure is an important mechanism in this regard.

**FIGURE 6:**
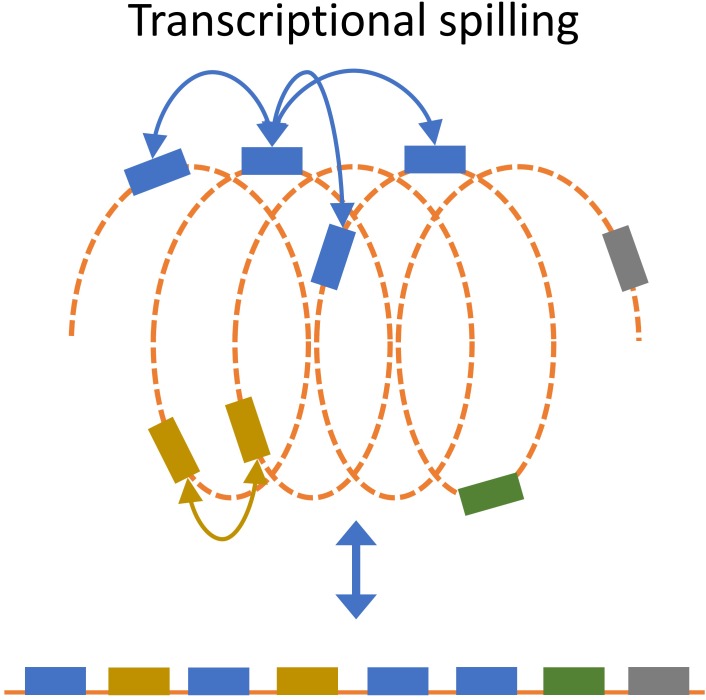
Transcriptional spill. Recruitment of RNAP to a gene will increase the likelihood that spatially close genes will interact with RNAP. Transcriptional spill is not apparent from the uncoiled linear organization of genes (bottom) but becomes apparent when considering spatial organization.

Transcription will recruit RNAP through increased association with the promoter, which will increase the local RNAP concentration thereby increasing the likelihood that nearby genes will interact with the RNAP (**Figure 6**; Rogozin et al., 2002; Nudler, 2009; Svetlov and Nudler, 2013; Wang et al., 2013a). It has been shown that gene expression is position-dependent (Bryant et al., 2014; Sauer et al., 2016). By inserting a reporter gene cassette at different sites on the *E. coli* chromosome, Bryant et al. (2014), observed ∼300 fold differences in expression (Bryant et al., 2014). Short- and long-range autocorrelation patterns as a function of the genomic distance between genes has also been observed (Jeong et al., 2004; Willenbrock and Ussery, 2004). The structure-function relationship has been used to develop a model that predicts the local three-dimensional structure of the nucleoid based on transcription data, but the extent and potential beneficial role of this spilling remains unclear (Jeong et al., 2004; Xiao et al., 2011, 2017).

One could imagine that extensive recruitment of RNAP by a highly transcribed gene can increase the expression of nearby genes (in 3D) through transcriptional spilling. This speculative concept is supported by evidence for low transferability of highly transcribed genes, since DNA with high RNAP recruitment would have a higher rate of spilling on nearby genes (Park and Zhang, 2012). This is further supported by observed proportional relation between contact frequencies and transcriptional level, which suggest that highly transcribed genes are organized such that the interact more often (Lioy et al., 2018). As such, the optimal position for sustainable integration of the foreign DNA might depend on its expression level. Gene insertion hotspots have been found in many bacterial species (Oliveira et al., 2017), and it would be interesting to investigate whether or not there is a higher level of foreign DNA integration at regions with low demand for structural integrity. By extension, less integration of foreign DNA would be expected to occur close to highly expressed genes due to higher demand for structural integrity.

Nucleoid structure preservation might further explain the accumulation of pseudo- and phantom-genes in bacteria. Can accumulation of redundant or non-coding genes be beneficial to bacteria in certain cases if a specific spatial position of genes relative to each other is favorable? Such non-functioning DNA may be preserved to keep a distinct structure and intergenic distance – a register – that is required for optimal fitness. Nucleoid spatial structure could generate synergy between strong promoters in spatial proximity, as observed at rRNA operons during exponential growth (Cagliero and Jin, 2013). Through these mechanisms, the three-dimensional structure of the nucleoid would impact the ability of bacteria to adapt and respond quickly to changes in the environment, since the specific spatial context of a gene might impact the expression level.

It is well established in eukaryotes that spatiality is critical for optimal regulation of genes and chromosomal structure (Lanctôt et al., 2007). Although bacterial chromosomes are smaller and arguably less complex compared to eukaryotes, tight control of nucleoid architecture is still required. Particularly during rapid growth where nucleoid replication and segregation happens simultaneously with up to >16 copies of origin-proximal regions. Understanding how chromosomal architecture influences DNA functionality and evolution will be of major importance not only for our basic understanding of genome evolution but also for rational design of novel bacterial strains for synthetic biology purposes and biotechnological production.

Our understanding of nucleoid evolution is still incomplete, in particular with respect to the mechanisms governing sustainable integration of horizontally acquired DNA. Evolution has no endgame, but the life of many prokaryotes has been highly optimized for adaptation to changing environments. Modulation of nucleoid structure through the action of NAPs, and the accumulation of pseudo- and phantom genes is therefore expected to strongly contribute to the evolutionary arms race.

## Author Contributions

TK, CK, and JM-J wrote the manuscript and approved its final version.

## Conflict of Interest Statement

The authors declare that the research was conducted in the absence of any commercial or financial relationships that could be construed as a potential conflict of interest.
